# Intermittent fasting and changes in clinical risk scores: Secondary analysis of a randomized controlled trial

**DOI:** 10.1016/j.ijcrp.2023.200209

**Published:** 2023-09-11

**Authors:** Benjamin D. Horne, Jeffrey L. Anderson, Heidi T. May, Viet T. Le, Tami L. Bair, Sterling T. Bennett, Kirk U. Knowlton, Joseph B. Muhlestein

**Affiliations:** a Intermountain Medical Center Heart Institute, Salt Lake City, UT, USA; b Division of Cardiovascular Medicine, Department of Medicine, Stanford University, Stanford, CA, USA; c Cardiology Division, Department of Internal Medicine, University of Utah, Salt Lake City, UT, USA; d Rocky Mountain University of Health Professions, Provo, UT, USA; e Intermountain Central Laboratory, Intermountain Medical Center, Salt Lake City, UT, USA; f Department of Pathology, University of Utah, Salt Lake City, UT, USA; g Division of Cardiovascular Medicine, Department of Medicine, University of California San Diego, La Jolla, CA, USA

**Keywords:** Therapeutic fasting, Intermountain risk score, IMRS, ICHRON, Pooled cohort risk equations

## Abstract

**Background:**

Intermittent fasting may increase longevity and lower cardiometabolic risk. This study evaluated whether fasting modifies clinical risk scores for mortality [i.e., Intermountain Mortality Risk Score (IMRS)] or chronic diseases [e.g., Pooled Cohort Risk Equations (PCRE), Intermountain Chronic Disease score (ICHRON)].

**Methods and results:**

Subjects (N = 71) completing the WONDERFUL trial were aged 21–70 years, had ≥1 metabolic syndrome criteria, elevated cholesterol, and no anti-diabetes medications, statins, or chronic diseases. The intermittent fasting arm underwent 24-h water-only fasting twice-per-week for 4 weeks and once-per-week for 22 weeks (26 weeks total). Analyses examined the IMRS change score at 26 weeks vs. baseline between intermittent fasting (n = 38) and *ad libitum* controls (n = 33), and change scores for PCRE, ICHRON, HOMA-IR, and a metabolic syndrome score (MSS). Age averaged 49 years; 65% were female. Intermittent fasting increased IMRS (0.78 ± 2.14 vs. controls: −0.61 ± 2.56; p = 0.010) but interacted with baseline IMRS (p-interaction = 0.010) to reduce HOMA-IR (but not MSS) more in subjects with higher baseline IMRS (median HOMA-IR change: fasters, −0.95; controls, +0.05) vs. lower baseline IMRS (−0.29 vs. −0.32, respectively). Intermittent fasting reduced ICHRON (−0.92 ± 2.96 vs. 0.58 ± 3.07; p = 0.035) and tended to reduce PCRE (−0.20 ± 0.22 vs. −0.14 ± 0.21; p = 0.054).

**Conclusions:**

Intermittent fasting increased 1-year IMRS mortality risk, but decreased 10-year chronic disease risk (PCRE and ICHRON). It also reduced HOMA-IR more in subjects with higher baseline IMRS. Increased IMRS suggests fasting may elevate short-term mortality risk as a central trigger for myriad physiological responses that elicit long-term health improvements. Increased IMRS may also reveal short-term fasting-induced safety concerns.

## Highlights

• Clinical risk scores were evaluated in subjects participating in the 6-month WONDERFUL trial.
• Intermittent fasting increased a mortality risk score, but decreased 10-year chronic disease risk scores.
• Fasting reduced insulin resistance more in subjects with higher baseline mortality risk.
• Fasting may elicit physiologic mechanisms that improve health by increasing mortality risk.
• Clinically, safety and benefits should be considered by people engaging in intermittent fasting.

## Graphical abstract

**Figure:**
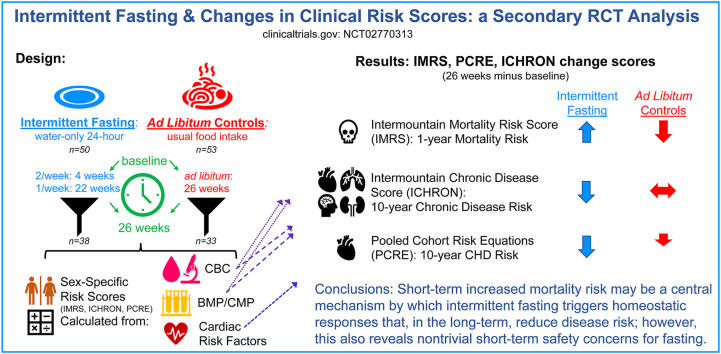

## 1 Introduction

Intermittent fasting is a term encapsulating multiple dietary regimens in which people deliberately cease energy intake for some daily length of time (e.g., 16, 18, or 24 h) combined with one of various weekly frequencies (e.g., every day, twice per week, or once per week). Myriad studies of intermittent fasting [[1], [2], [3]], including studies of the fasting regimen time-restricted eating [4,5], and studies of religious fasting [6,7], report that fasting reduces risk factors for cardiovascular and metabolic diseases. Observational studies report associations of periodic fasting with lower risks of disease diagnoses, including coronary artery disease (CAD) [8,9], diabetes [8,9], and heart failure (HF) [10]. A popular claim is that fasting improves longevity, but associations of periodic fasting with lower risk of mortality in cardiovascular patients and lower risk of a composite of mortality and hospitalization in patients with severe acute respiratory syndrome coronavirus 2 are shown only by observational cohort studies [10,11]. No randomized controlled trial examined intermittent fasting for delaying mortality or preventing cardiac diagnoses.

## 2 Theory

### 2.1 Intermittent fasting and longevity

The most commonly examined outcomes in interventional trials of intermittent fasting are weight loss and other body composition measures [1,2,6,7]. Evidence shows, though, that fasting produces cardiometabolic health benefits regardless of the amount of weight lost or even if no weight change occurs [4,5]. These weight change-independent effects are potentially powerful benefits that can be realized over the lifespan, even with low-dose intermittent fasting, and lead to greater longevity as indicated by observational longitudinal studies of periodic fasting [10,11]. Randomized controlled trials of a dietary regimen like intermittent fasting and the outcome of longevity are difficult, though, because of the long interventional period that is required in which adherence to a fasting intervention is likely to be very low and difficult to track, and because of the very long follow-up time necessary to observe mortality events. It is possible, though, to examine a risk score developed to predict mortality risk as a surrogate measure of mortality in a shorter-term randomized trial of intermittent fasting.

### 2.2 The Intermountain Mortality Risk Score (IMRS)

IMRS is a widely-validated quantitative longevity prediction tool computed from objective, standardized clinical laboratory factors in the complete blood count (CBC) and basic metabolic profile (BMP) [[12], [13], [14], [15], [16], [17], [18], [19], [20]]. IMRS was developed for clinical mortality risk assessment in outpatients and inpatients [12], and has a similar to superior ability to predict mortality compared to comorbidity scores [15,16,20]. IMRS also predicts the diagnosis of major causes of death, including HF, myocardial infarction (MI), CAD, and diabetes [21]. IMRS is in use at Intermountain Health for clinical decision support, is under investigation for such use elsewhere [15,20], and was shown to guide clinical decision-making in HF inpatients in which a risk score-driven precision medicine process showed simultaneous reductions in risks of hospital readmission and mortality [18]. IMRS is a dynamic biomarker of modifiable risk for which longitudinal reductions or elevations reveal, respectively, decreases or increases in the risk of mortality [19,22]. Such IMRS changes reflect alterations in physiological states related to improvements or declines in health and do not necessarily indicate an irreversible condition.

### 2.3 Study objectives

Because the greatest benefit of interest from intermittent fasting is longevity extension and IMRS was developed to predict risk of mortality, this study evaluated whether intermittent fasting modified the 1-year IMRS over a 26-week (6-month) period in a secondary analysis of a randomized controlled trial. Secondarily, the study examined whether intermittent fasting altered the metabolic status of subjects differentially according to baseline risk score levels or if fasting changed scores that predict 10-year chronic disease: the Pooled Cohort Risk Equations score (PCRE) and Intermountain Chronic Disease score (ICHRON) [[23], [24], [25]].

## 3 Materials and methods

### 3.1 Trial randomization, population, and ethics

The broader environment of the Weekly ONe-Day watER-only Fasting interventionaL (WONDERFUL) trial (registration: clinicaltrials.gov, NCT02770313) was that it was a single-site randomized controlled trial of an intermittent fasting regimen using low frequency once-per-week repetition of 24-h water-only fasting compared to an *ad libitum* diet control group [26]. The primary findings of the larger trial were previously published, including that intermittent fasting reduced homeostatic model assessment of insulin resistance (HOMA-IR) and a metabolic syndrome score (MSS), despite that weight was not significantly reduced for intermittent fasting compared to controls [26]. The trial CONSORT diagram is available in that publication [26]. An *a priori* planned analysis designated in the trial protocol was to evaluate whether IMRS, an efficient risk score for 1-year mortality, was changed by the 26-week intermittent fasting intervention, and this hypothesis test is reported herein as the primary outcome. The analyses of changes in 10-year coronary heart disease risk encapsulated by PCRE and of potentially other summary risk metrics were also pre-specified in the protocol [ICHRON was not derived to predict 10-year chronic disease incidence until 2018 [24,25]]. The research protocol specifying the secondary analyses of IMRS and the other risk scores was approved by the Intermountain Health Institutional Review Board (IRB number 1050163) and registration at clinicaltrials.gov (NCT02770313) was completed in 2016 prior to the beginning of subject recruitment. Subjects provided written informed consent to participate in the trial during an enrollment phase from November 2016–August 2019 and the final subject follow-up occurred on February 19, 2020 (16 days before research shut-down due to coronavirus disease 2019).

Randomization utilized a permuted block approach with 1:1 parallel arm allocation using sequentially numbered envelopes generated by a trained statistician (Dr. May). Investigators and the statistical analyst, but not subjects, were blinded to randomization allocation. The 24-h water-only intermittent fasting regimen included an initial frequency of twice per week fasting on non-consecutive days for a period of 4 weeks. Following this “loading dose,” fasting transitioned to one day per week for a period of 22 weeks, for a total intervention period of 26 weeks. *Ad libitum* eating was followed by subjects in the fasting arm on non-fasting days and by those in the control arm on all days over the 26 weeks of the trial. Adherence was monitored using a diary for the 38 subjects randomized to the fasting intervention and, as previously reported [26], averaged 95 ± 12% at the 26-week study end with only two subjects reporting adherence <80% (70% and 37%).

Potential study subjects were recruited by direct contact of prior Intermountain patients whose electronic health records revealed that it was probable that they met study inclusion and exclusion criteria. They were also recruited through advertising the study to patients at hospital- or community-based outpatient clinics and to community-dwelling individuals through traditional and social media and via health fairs. Individuals self-selected to contact the study personnel to explore enrollment in part because they were aware of their elevated cardiac risk factors, including cholesterol levels, blood pressures, weight, and glycemic status, and often enrolled because they were already familiar with intermittent fasting and interested in the intervention.

Subjects were enrolled and followed up in the research clinic at Intermountain Medical Center by clinical research coordinators. They were included in the trial if they had a modest elevation of low-density lipoprotein cholesterol (LDL-C) and one or more factors of the metabolic syndrome, including: 1) triglycerides ≥150 mg/dL or use of medication to reduce triglycerides, 2) HDL-C <50 mg/dL for females or <40 mg/dL for males, 3) fasting glucose >100 mg/dL, 4) waist circumference ≥35 inches for females, ≥40 inches for males, or BMI >25 kg/m^2^, or 5) systolic blood pressure ≥130 mmHg, diastolic ≥85 mmHg, or antihypertensive use [27]. Other inclusion criteria were: males and non-pregnant females aged 21–70 years who were not taking statins or anti-diabetic medications, and exclusions were: prior diagnosis of a chronic disease such as type 1 diabetes, coronary heart disease, cancer, cerebrovascular disease, chronic kidney disease, peripheral vascular disease, chronic obstructive pulmonary disease, dementia, eating disorder, solid organ transplantation, or immunodeficiency [26]. Physical exams, laboratory testing, review of fasting logs for adherence, documentation of dietary changes, and completion of study questionnaires were conducted at four study visits at baseline, 4 weeks, 13 weeks, and 26 weeks. Fifty subjects were randomized to intermittent fasting and n = 38 (76%) completed the 26-week regimen, while 53 were randomized to the control arm and 33 (62%) completed the study. As reported previously [26], 8 subjects withdrew from the control arm who expressed a discomfort with that assignment while nobody withdrawing from the intervention arm provided that as the reason; otherwise, drop-outs were similar between the two arms. Based on study follow-up, health system records, Utah death certificates, and US Social Security Administration data, no subjects enrolled in the trial died during their six-month participation.

### 3.2 Study outcome measures

The CBC and BMP data utilized to compute 1-year IMRS values were collected through study laboratory testing at the four study visits using clinical hematology and chemistry analysis of fresh blood samples in the central clinical laboratory at Intermountain Medical Center (Salt Lake City, UT). The sex-specific calculations of IMRS using age, CBC factors, and BMP parameters for prediction of 1-year mortality were previously published [12]. The CBC panel included the red blood cell count, hematocrit, hemoglobin, white blood cell count, platelet count, mean corpuscular volume (MCV), mean corpuscular hemoglobin (MCH), mean corpuscular hemoglobin concentration (MCHC), red cell distribution width, and mean platelet volume (MPV). The BMP panel was a subset of parameters from the comprehensive metabolic profile, including glucose, creatinine, blood urea nitrogen, sodium, potassium, chloride, calcium, and bicarbonate. Age was calculated based on the baseline study visit and subject-reported birthdate, and sex was self-reported by each subject.

Secondary endpoints (i.e., PCRE, ICHRON, HOMA-IR, and MSS) used data from clinical laboratory testing, including insulin, total cholesterol, LDL-C, high-density lipoprotein cholesterol (HDL-C), and triglycerides from the four trial visits. They also used physical examination data that measured systolic and diastolic blood pressures, weight, and waist circumference. Race, history of smoking, prior diabetes diagnosis, and anti-hypertensive use were also documented from subject report of demographics and medical history.

PCRE, a predictor of 10-year atherosclerotic cardiovascular disease risk, was calculated using previously reported methodology for race- and sex-specific scoring of age, total cholesterol, HDL-C, systolic blood pressure, anti-hypertensive medication, smoking history, and diabetes diagnosis [23]. ICHRON, a predictor of 10-year risk of the first diagnosis of 10 chronic cardiovascular (i.e., CAD, MI, HF, atrial fibrillation, stroke, dementia, chronic kidney disease, peripheral vascular disease), metabolic (i.e., type 2 diabetes), and pulmonary diseases (i.e., chronic obstructive pulmonary disease), was calculated based on prior literature using sex-specific weightings of age, CBC factors, and comprehensive metabolic panel parameters (i.e., including albumin, bilirubin, total protein, alkaline phosphatase, alanine transaminase, and aspartate aminotransferase and the components in the BMP) [24,25]. Glucose and insulin levels were used to calculate HOMA-IR using the standard approach: (glucose * insulin)/405 where glucose is measured in mg/dL and insulin in mIU/L. The MSS was calculated using sex-specific weightings of waist circumference, HDL-C, triglycerides, blood pressures, and glucose, as done previously [28,29].

### 3.3 Statistical analyses

Baseline characteristics of study subjects are reported for each randomization arm by categories of change in IMRS across the 26 weeks of the trial, with IMRS categorized as above and below the median change (with the median being determined separately for females and males but regardless of randomization arm). Comparisons between these groups were analyzed by Student's T-test or the chi-square test, as appropriate. Changes in IMRS, PCRE, ICHRON, HOMA-IR, and MSS were defined as the change scores for each variable's 26-week value minus its baseline value. The assumption of normality for the distribution of data in outcome variables was examined using the Kolmogorov-Smirnov test.

Change scores for IMRS, PCRE, and ICHRON were evaluated between intermittent fasting and control arms using general linear models with adjustment for sex because each of these scores is calculated with sex-specific parameter weightings that places the risk score ranges (and, thus, their change scores) for females and males on a different scale. Changes in HOMA-IR and MSS were evaluated between intermittent fasting and control arms using the Mann-Whitney nonparametric approach due to non-normal data distributions. All analyses of randomization arms used the intent-to-treat approach and subjects with missing data were excluded from analyses (which primarily meant that the 32 subjects with no 26-week final visit were excluded). General linear models with adjustment for sex were further used to examine statistical interactions of randomization group for effect modification by baseline IMRS, PCRE, or ICHRON for changes in IMRS, PCRE, or ICHRON. For changes in HOMA-IR and MSS, which were non-normally distributed, quantile regression with adjustment for sex was used for interaction analyses of randomization arm with baseline IMRS, PCRE, or ICHRON. The trial was originally powered to detect a change in LDL-C, as previously described [26], with IMRS and PCRE designated as secondary endpoints. A p-value at p ≤ 0.05 was defined as statistically significant for the primary outcome of IMRS change and other outcomes were not adjusted for multiple comparisons and, thus, are considered exploratory. Study analyses utilized SPSS v26.0 (IBM SPSS, Armonk, NY).

## 4 Results

*4.1* A sample size of N = 69 subjects (67%) had data available to compute IMRS (and ICHRON) and primary analyses focused on this population, with n = 71 (69%) having data available to compute PCRE. Baseline characteristics stratified by randomization arm and by the 26-week change in IMRS are provided in Table 1. Age averaged 49.4 years and almost 65% of subjects were female. The baseline IMRS averaged 4.97 ± 2.46 in females and 6.45 ± 2.84 in males, both of which are low-risk levels [upper limit of low-risk: 8 in females, 10 in males [12]]. Generally, few differences in baseline variables were found based on how IMRS changed, with the exceptions being (Table 1) that individuals randomized to fasting who had IMRS changes above the median had lower baseline diastolic blood pressures and lower baseline MSS, while those in the control group with higher IMRS changes had higher baseline glucose.

**Table 1:** Baseline characteristics by randomization arm and category of IMRS change. Baseline CBC and metabolic profile components not listed here were not different between IMRS change groups and are not shown for table simplicity.

	**Randomization: Intermittent Fasting**		**Randomization:*Ad Libitum*Controls**	
**Baseline**	**26-Week Change**	**26-Week Change**	**26-Week Change**	**26-Week Change**
**Characteristic**	**in IMRS ≤ Median**^a^	**in IMRS ≥ Median**^a^	**in IMRS ≤ Median**^a^	**in IMRS ≥ Median**^a^
Sample Size	13	23	21	12
Age (years)	50.0 ± 10.0	50.4 ± 13.4	47.5 ± 10.8	50.5 ± 8.5
Sex (Female)	53.8% (7)	65.2% (15)	66.7% (14)	75.0% (9)
Race (nonwhite)	0% (0)	8.7% (2)	0% (0)	0% (0)
Weight (kg)	109 ± 17	97 ± 30	102 ± 24	98 ± 14
Waist circumf. (cm)	113 ± 12	104 ± 19	109 ± 15	105 ± 12
SBP (mmHg)	131 ± 16	125 ± 10	128 ± 13	133 ± 6
DBP (mmHg)	87.3 ± 9.9	79.3 ± 6.8†	80.5 ± 9.2	81.1 ± 10.8
Type 2 Diabetes	0% (0)	0% (0)	4.8% (1)	0% (0)
Smoking history	0% (0)	0% (0)	0% (0)	0% (0)
Anti-hypertensive use	0% (0)	0% (0)	0% (0)	0% (0)
Insulin (mIU/L)	11.8 ± 5.0	9.5 ± 5.0	12.2 ± 6.5	12.4 ± 7.2
Glucose (mg/dL)	100 ± 18	90 ± 13	89 ± 8	96 ± 10§
HOMA-IR	2.87 ± 1.14	2.20 ± 1.41	2.83 ± 1.64	2.95 ± 1.98
MSS	2.34 ± 4.28	−0.50 ± 3.37‡	0.84 ± 3.50	0.56 ± 3.27
Total cholesterol (mg/dL)	199 ± 25	194 ± 28	199 ± 24	216 ± 25
LDL-C (mg/dL)	123 ± 24	124 ± 19	126 ± 18	136 ± 14
HDL-C (mg/dL)	44.9 ± 10.5	47.0 ± 10.5	43.7 ± 9.9	51.3 ± 15.0
Triglycerides (mg/dL)	154 ± 72	117 ± 78	148 ± 74	145 ± 70
IMRS (1-year risk of all-cause mortality)				
Females	5.86 ± 2.27	4.13 ± 2.39	5.21 ± 2.16	4.78 ± 3.07
Males	8.00 ± 2.76	6.63 ± 2.13	7.71 ± 2.29	4.33 ± 2.31
PCRE (10-year risk of atherosclerotic cardiovascular disease)				
Females	−25.3 ± 5.2	−25.6 ± 5.4	−24.4 ± 4.7	−25.9 ± 3.5
Males	−0.1 ± 0.2	−0.1 ± 0.2	−0.1 ± 0.1	−0.1 ± 0.1
ICHRON (10-year risk of chronic cardiovascular, metabolic, or pulmonary diseases)				
Females	16.1 ± 2.0	13.1 ± 4.1	11.2 ± 5.2	8.1 ± 4.9
Males	10.0 ± 5.1	9.9 ± 7.2	14.2 ± 5.8	8.7 ± 2.3

a Median IMRS change was 1.0 for females and −1.0 for males; Comparison between IMRS change groups: †p = 0.007 and ‡p = 0.034 in subjects randomized to intermittent fasting, §p = 0.037 in subjects randomized to *ad libitum* control.

**Fig. 1:**
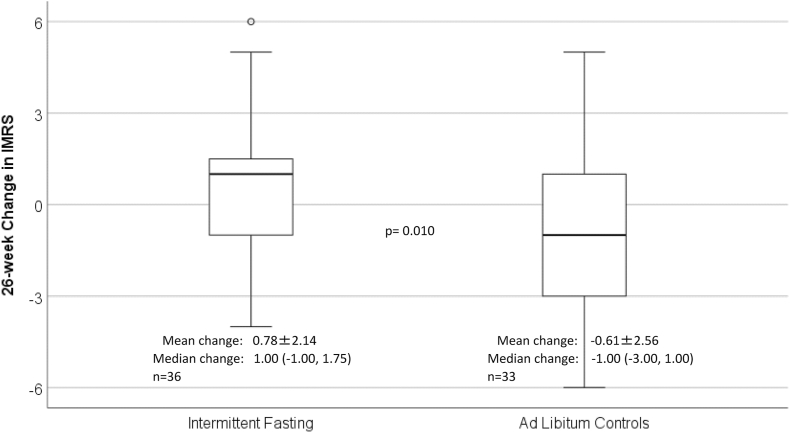
Boxplots showing the 26-week change scores for IMRS. Bold lines in the boxes are median IMRS, the top and bottom box limits are 25th and 75th percentiles, the whiskers extend to 1.5 times box height, open circles outside the whiskers are outlying values > 1.5 times box height, and an asterisk outside the whiskers is an outlying value > 3.0 times box height. Both the mean IMRS change with the standard deviation (SD) and median IMRS change with interquartile range are provided for each randomization group under the relevant boxplot, along with the sample size for each.

**Fig. 2:**
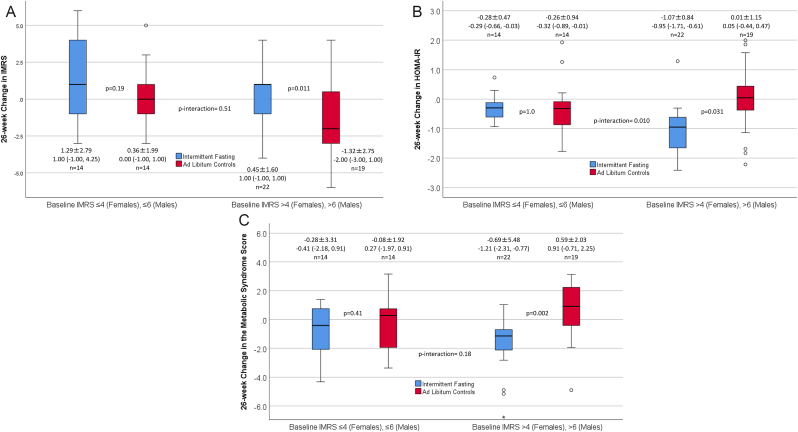
These boxplots show 26-week changes in: (**a**) IMRS, (**b**) HOMA-IR, and (**c**) MSS between intermittent fasting and control groups stratified by baseline IMRS below the median or baseline IMRS above the median (medians were 4 for females and 6 for males). Effect modification by baseline IMRS was found for the impact of intermittent fasting on changes in HOMA-IR but not on changes in IMRS or MSS, with more profound changes when subjects' baseline IMRS was above the median. Boxplot symbols are as described in Fig. 1 legend; data provided under or above each boxplot are the mean change ±SD, median change (interquartile range), and sample size for each group.

*4.2* Over the course of the 26-week intervention, the primary outcome of this analysis of the risk of mortality measured by the dynamic laboratory-based IMRS was unexpectedly increased by intermittent fasting (p = 0.010) compared to *ad libitum* controls (Fig. 1). This IMRS increase was not modified by baseline IMRS (Fig. 2A), with numerically similar IMRS increases in subjects in the intermittent fasting arm regardless of baseline IMRS being above or below the median (>4 vs. ≤4 for females, >6 vs. ≤6 for males). IMRS was unchanged in controls who had lower baseline IMRS (≤4 for females, ≤6 for males) but controls with higher baseline IMRS (>4 for females, >6 for males) appeared to have some decline perhaps due to regression to the mean.

*4.3* However, previously-reported [26] intermittent fasting-related changes in HOMA-IR did interact with baseline IMRS (Fig. 2B) in which a significantly greater decline in HOMA-IR (p-interaction = 0.010) was found in fasting subjects who had a higher baseline IMRS {median HOMA-IR change: intermittent fasting, −0.95 [interquartile range (IQR): −1.71, −0.61] vs. *ad libitum* controls, 0.05 [IQR: −0.44, 0.47]}. No apparent change in HOMA-IR was recorded in those with lower baseline IMRS [median HOMA-IR change: intermittent fasting, −0.29 (IQR: −0.66, −0.03) vs. *ad libitum* controls, −0.32 (IQR: −0.89, −0.01)]. An interaction of randomization arm with baseline IMRS was not found for changes in MSS (Fig. 2C).

**Fig. 3:**
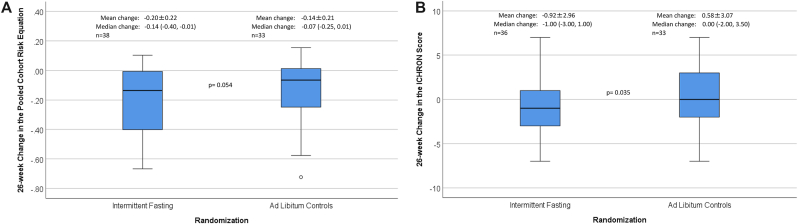
Boxplots comparing 26-week change scores for: (**a**) PCRE, and (**b**) ICHRON, showed a trend toward decreased PCRE and significantly decreased ICHRON for intermittent fasting compared to controls. Boxplot symbols are as described in Fig. 1 legend; data provided above each boxplot are the mean change ±SD, median change (interquartile range), and sample size for each group.

*4.4* For other risk scores, however, results were consistent with the expected effect of intermittent fasting in reducing both PCRE and ICHRON. Changes in the 10-year risk of atherosclerotic cardiovascular disease as measured by 26-week change in PCRE (Fig. 3A) exhibited a trend to being reduced by intermittent fasting compared to *ad libitum* controls (p = 0.054). An interaction of intermittent fasting with baseline PCRE was found (p-interaction = 0.017) in which subjects with higher baseline PCRE (i.e., baseline PCRE above the median: ≥ −24.0 for females, ≥ −0.1 for males) had greater declines in PCRE over the 26-week trial (PCRE change: intermittent fasting, −0.243 ± 0.246, vs. controls, −0.115 ± 0.189, p = 0.11) than in those with baseline PCRE below the median (<−24.0 for females, < −0.1 for males) (PCRE change: intermittent fasting, −0.148 ± 0.193, vs. controls, −0.159 ± 0.228, p = 0.86). However, no interactions of fasting with baseline PCRE were found for changes in HOMA-IR (median HOMA-IR change: in lower baseline PCRE: intermittent fasting, −0.716, vs. controls, −0.314, p = 0.053; in higher baseline PCRE: intermittent fasting, −0.789, vs. controls, 0.184, p = 0.012; p-interaction = 0.69) or changes in MSS (median MSS change: in lower baseline PCRE: intermittent fasting, −0.584 vs. controls, 0.495, p = 0.12; in higher baseline PCRE: intermittent fasting, −1.07, vs. controls, 0.69, p = 0.002; p-interaction = 0.45).

**Fig. 4:**
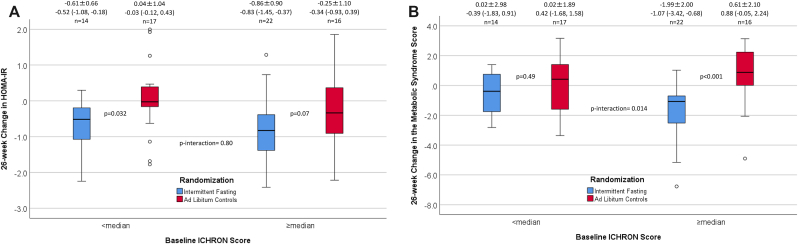
These boxplots show 26-week changes in: (**a**) HOMA-IR, and (**b**) MSS between intermittent fasting and control groups stratified by baseline ICHRON below or above the median (medians: 13 for females; 10 for males). Effect modification by baseline ICHRON was found for the impact of intermittent fasting on MSS changes but not HOMA-IR changes, with more profound changes when baseline ICHRON was above the median. Boxplot symbols are as described in Fig. 1 legend; data above each boxplot are the mean change ±SD, median change (interquartile range), and sample size for each group.

Intermittent fasting significantly reduced the 10-year risk of chronic cardiovascular, metabolic, and pulmonary diseases as encapsulated by the 26-week change in ICHRON (Fig. 3B) when compared to *ad libitum* controls (p = 0.035). No interaction for change in ICHRON was found of fasting with baseline ICHRON (above vs. below the median: ≥13 vs. <13 for females, ≥10 vs. <10 for males). The ICHRON changes in subjects with lower baseline ICHRON were: intermittent fasting, 0.07 ± 3.39, vs. 1.53 ± 2.98 in controls (p = 0.22), and in those with higher baseline ICHRON were: intermittent fasting, −1.55 ± 2.54, vs. −0.44 ± 2.92 in controls (p = 0.16) (p-interaction = 0.82). An interaction of higher baseline ICHRON (≥13 for females, ≥10 for males) with intermittent fasting was found (Fig. 4) for the change in MSS, but not for the change in HOMA-IR. Changes of individual components of the CBC and metabolic profiles are shown in Supplemental Table S1.

*4.5* Adverse events were previously reported [26], and were mostly minor. All of the following minor events were in the fasting arm: at 4 weeks, a total of one report was made for each of the following: constipation, insomnia, light-headedness, muscle tension, sweating, nausea, vomiting, and low energy or weakness, while two subjects had dizziness at 4 weeks and one at 13 weeks, two reported headache at 4 weeks and one at 13 weeks, one individual had intolerance to cold at 13 weeks, one subject experienced nausea at 13 weeks, and two reported diarrhea at 4 weeks, one at 13 weeks, and one at 26 weeks. Adverse events requiring physician attention that were changes from baseline were in the fasting arm and included a subject with high troponin I (the elevated troponin resolved within 24 h and was adjudicated to not be of cardiac origin), one with high bilirubin, and one with low sodium at 13 weeks, and a low potassium at 26 weeks. Edema was reported by 1 subject in the control arm at 4 weeks. No reports of excessive hunger, syncope, falls, or hypoglycemia were recorded and no deaths or new diagnoses of diabetes, CAD, MI, or stroke were found [26].

## 5 Discussion

### 5.1 Summary

In secondary analyses of a randomized controlled trial, low-frequency intermittent fasting increased IMRS, a clinical risk prediction tool that encapsulates the 1-year risk of mortality. Fasting concomitantly decreased PCRE and ICHRON, scores that both describe 10-year risks of chronic disease onset. Further, intermittent fasting interacted with high baseline IMRS in modifying the effect of fasting on metabolic outcomes such as reducing HOMA-IR substantially more in subjects with baseline IMRS above the median, but an interaction of IMRS and fasting was not confirmed in MSS reduction.

### 5.2 Intermittent fasting and longevity

A common claim of health gurus who highlight intermittent fasting in social media posts, product advertisements, and weight loss books is that fasting increases longevity. Almost inevitably they cite animal study results and anecdotal human cases to validate this claim. Scientific evaluations of fasting and longevity in humans are very limited, though, with a few epidemiologic studies reporting that individuals who routinely engaged in periodic fasting for decades survived longer than their peers [10,11]. The majority of scientific evidence in humans for longevity extension by fasting relies on extrapolations of controlled trials evaluating cardiometabolic risk factors or observational studies examining the risk of major morbidities [[1], [2], [3], [4], [5], [6], [7], [8], [9]]. This exciting and growing body of literature does generally indicate that the various fasting regimens may provide additional benefit beyond established principles of a healthy diet [30].

Some observational fasting studies evaluated short-term mortality risk, including comparisons of deaths at the time of Ramadan to deaths occurring the rest of the year [31,32]. Mortality was higher during the Ramadan fasting period [31,32]. This suggests that during a period when fasting is being practiced actively that physiological stressors may be at work that, in measured doses for those who can manage the stress, likely trigger the reported health benefits of fasting (a process sometimes called “hormesis”). Those results also suggest that the stress of fasting may be too great for some people, perhaps those who are already experiencing health challenges such as advanced comorbidities, frailty, or uncontrolled metabolic disease, and this raises important safety concerns regarding intermittent fasting. The consideration of mortality and other severe adverse events is of paramount importance in part due to the ease with which fasting may be tried without guidance or prescription and in part because of the widespread adoption of or individual experimentation with fasting in the general public. Thus, due to the potential for increased mortality risk by fasting, the use of fasting for health improvement should include consideration of safety [31,32], especially for people with existing chronic diseases [33].

### 5.3 Implications of these findings

This study reports an increase in a risk score that predicts mortality and supports the need for safety evaluation based on changes in IMRS, a powerful quantitative, objective, temporally-dynamic risk score [[12], [13], [14], [15], [16], [17], [18], [19], [20], [21]]. When IMRS is measured multiple times longitudinally, those serial measurements were found previously to provide independent risk information [19,22]. In the present study, IMRS was increased by intermittent fasting over a 6-month period while, simultaneously, two risk scores that predict chronic diseases were reduced. This includes the decrease of ICHRON that, like IMRS, uses the CBC and metabolic profile but weights their components to predict chronic disease onset rather than mortality [24,25]. Because ICHRON uses a few different components than IMRS, a possibility exists that these laboratory variables are differentially affected by fasting, but these are minimal (e.g., use of chloride instead of sodium that are collinear variables, or use of albumin, total protein, and alkaline phosphatase that add only limited risk information for death or disease onset) [12,24].

The apparent discrepancy of IMRS elevation and declines in PCRE and ICHRON may more likely implicate elevated mortality risk as a central mechanism for evoking the multiplicity of health benefits attributed to it, but this may be a short-term or transitory effect that eventually resolves. The stress that fasting places on the body may cause the physiologic changes that lead to mortality to be manifest and, thereby, trigger a cascade of survival responses. The changes in IMRS likely reflect this by encapsulating changes in the components of the CBC and BMP laboratory panels. That is, fasting may cause a departure from homeostasis that cells, tissues, and organs recognize as a threat and that mobilizes self-defense mechanisms and functional optimization responses. This includes tapping stored energy sources through ketosis [[34], [35], [36]], reducing insulin resistance [3,26], minimizing inflammation [[37], [38], [39]], bolstering the immune system [37], increasing oxygen carrying capacity [40], inducing mitochondrial uncoupling [41,42], activating and enhancing autophagy [37,43,44], degrading dysfunctional mitochondria [45], modifying the microbiome [46], and potentially affecting other mechanisms. These responses limit processes that lead to chronic diseases and, eventually, should increase longevity. The simultaneous declines in PCRE and ICHRON due to intermittent fasting suggest that future diagnoses of chronic diseases will be reduced and, thus, that this risk reduction would, over a period of years and decades, prevent long-term increases in IMRS or even reduce IMRS and that this should lead to lower lifetime risk of mortality compared to non-fasters, as some evidence suggests [10,11].

The proposal that the observed IMRS increase measures a trigger for intermediate- and long-term disease risk reduction and longevity improvement is, in part, suggested by the minimal clinical significance of the observed median 1.0 (mean: 0.78) increase in IMRS (theoretical IMRS range in females: −5 to 28; males: −1 to 28) [12]. Prior literature shows that increase represents a minor change in actual mortality [[12], [13], [14], [15], [16], [17], [18], [19], [20], [21]]. In particular, a study of the change score of IMRS from baseline to a second IMRS measurement 6–24 months later revealed an average IMRS increase of 1.6 and 2.0 for female and male patients, respectively (2.05–2.56 fold higher than the average IMRS change observed here), whose baseline IMRS values were in a low-risk range similar to the range of IMRS values in this WONDERFUL Trial study [19]. Over a 4-year follow-up period after the second post-6-month IMRS measurement, risk of mortality in that patient population had hazard ratios of 1.20 per +1 IMRS and 1.14 per +1 IMRS [19]. Based on those values, an estimation of the 4-year mortality risk for an average increase of 0.78 IMRS may be estimated at a hazard ratio of 1.16 for females and 1.11 for males, although the WONDERFUL Trial subjects were, further, free of chronic disease diagnoses and were not in need of patient care, placing them potentially at even lower risk. Further, the interaction of a higher baseline IMRS with intermittent fasting in more profoundly reducing HOMA-IR suggests that fasting more readily triggers health improvements in people whose homeostasis is already perturbed [47]. Conceivably, the intensity of a short-term IMRS increase causes gradations in the trigger of such a response, with people whose IMRS is already relatively elevated having a more profound response as IMRS rises due to fasting.

The fasting-induced changes in IMRS are similar to other interventions. For example, during and shortly after vigorous intensity aerobic exercise, there is an increased relative risk of acute cardiac events and sudden cardiac death, yet repeated aerobic exercise over the long-term reduces risk of cardiovascular morbidity and mortality [48]. Similarly, strength training involves short-term risks (e.g., elevated blood pressure, pain, and muscle weakness) but long-term cardiovascular benefits [48]. Travel to higher altitudes also involves some risks (e.g., acute mountain sickness), but physiological adaptation (i.e., acclimatization) can normalize and improve human performance [49].

### 5.4 Clinical implications

Finally, if intermittent fasting elevates short-term mortality risk, this is a clinical safety issue involving nontrivial adverse events. Intermittent fasting is effective for achieving certain health improvements such as weight loss, reduction of insulin resistance, improvement in glycemic control, and reduction in cardiovascular risk factors and it is generally safe [[1], [2], [3], [4], [5], [6], [7],^26^], but potential risks do exist [33]. Caution should be taken when someone initiates a fasting regimen, especially older people or those with chronic diseases, and the expected benefits of intermittent fasting should be weighed along with the potential risks. The physiologic stress of fasting may be excessive for people who are frail, carry a diabetes diagnosis, or have renal failure, stage IIIb-V chronic kidney disease, advanced cancer, active cancer treatment, chronic obstructive pulmonary disease, coronary disease, New York Heart Association class III/IV HF, prior stroke, prior MI, or other conditions [33]. The dosage of intermittent fasting should be chosen with care to achieve hormesis and avoid serious adverse effects. This is similar to dose titration of cardiovascular medications such as sodium-glucose cotransporter 2 inhibitors or beta-blockers that improves health but at high doses can, respectively, harm renal function or induce negative inotropic effects. Other clinical safety concerns exist (e.g., children should not fast for health purposes unless it is prescribed by a clinician; people with an eating disorder should not fast). Other fasting regimens that involve a shorter fasting length, such as the 16-h fast of time-restricted eating [[4], [5], [6], [7]], may be less challenging and have fewer potential safety issues than a 24-h fast, although the cardiometabolic and body composition benefits of the various fasting regimens may differ.

### 5.5 Limitations

This paper reports a secondary evaluation of prospectively-collected data in a randomized clinical trial whose original objective was not focused on IMRS, PCRE, or ICHRON. Although measured variables were not different between intervention and control arms [26], the trial randomization procedure may not have balanced all factors relevant to risk score analyses if some important confounder was not measured. Further, the trial sample size was not large, detailed dietary intake data were not collected, and the endpoints were not clinical events but risk scores for such events [26]. Also, participants were community-dwelling adults with some cardiometabolic risk factors but no cardiovascular diseases, cancer, respiratory conditions, or other major causes of death, and since most IMRS studies evaluated outpatients and inpatients [12,[14], [15], [16], [17], [18], [19]], the meaning of a 0.78 point IMRS increase for apparently healthy people and the generalizability of findings to other populations is unknown. Strengths of the study were the low-frequency 24-h fasting regimen that evoked improvements in cardiometabolic risk factors even when weight was not significantly decreased [26], and the *ad libitum* control of subjects utilizing their standard dietary choices that reflect real-world behaviors.

### 5.6 Conclusions

In secondary analyses of a randomized controlled trial, 26-week once-per-week water-only 24-h intermittent fasting increased the 1-year IMRS mortality risk score a clinically modest but statistically significant amount. In contrast, intermittent fasting decreased 10-year chronic disease risk captured by PCRE and ICHRON. Further, elevated baseline IMRS interacted with intermittent fasting to provoke a greater decline in HOMA-IR than for subjects with lower IMRS, suggesting that those with higher baseline IMRS received more cardiometabolic benefits. The short-term rise in IMRS may quantify a primary physiological trigger by which fasting activates short-term survival mechanisms and elicits long-term health improvements and longevity extension. Clinically, these findings suggest that intermittent fasting may involve non-trivial risks that must be considered and balanced with the potential benefits of fasting when individuals consider initiating an intermittent fasting regimen for health improvement, especially if they have existing morbidities. While intermittent fasting continues to be recommended, further research regarding intermittent fasting and IMRS is indicated, including whether IMRS continues to rise or plateaus and declines beyond 6 months of fasting and how much safety risk may be imbued by fasting regimens.

## Funding

This research was funded by two grants from the 10.13039/100008173Intermountain Research and Medical Foundation: grant number 759 (PI: BDH) and a supplemental unnumbered grant provided through the philanthropy of the Dell Loy Hansen Heart Foundation (PI: BDH). The funding source had no role in the design of the study; in the collection, analyses, or interpretation of data; in the writing of the manuscript, or in the decision to publish the results.

## Competing interests

BDH, HTM, and JLA are inventors of risk scores (including IMRS; not ICHRON) licensed by Intermountain to Alluceo and CareCentra. BDH is a member of the advisory boards of Opsis Health and Lab Me Analytics, a consultant to Pfizer regarding risk scores (funds paid to Intermountain), PI of a risk prediction grant from 10.13039/100004325AstraZeneca, and site PI of a grant funded by the Task Force for Global Health. KUK and BDH are site PIs of grants from the Patient-Centered Outcomes Research Institute and the NIH RECOVER initiative. The authors declare no other potential conflicts of interest.

## Data availability

The data underlying this article cannot be shared publicly due to United States privacy laws. The data and trial protocol will be shared on reasonable request to the corresponding author.

## Credit author statement

Benjamin Horne: Conceptualization, Methodology, Formal analysis, Investigation, Resources, Writing – original draft, Visualization, Supervision, Funding acquisition; Jeffrey Anderson: Conceptualization, Investigation, Writing – review & editing, Supervision, Funding acquisition; Heidi May: Methodology, Formal analysis, Resources, Data curation, Writing – review & editing, Visualization; Viet Le: Investigation, Resources, Writing – review & editing, Project administration; Tami Bair: Resources, Data curation, Writing – review & editing; Sterling Bennett: Methodology, Resources, Data curation, Writing – review & editing; Kirk Knowlton: Writing – review & editing, Supervision, Funding acquisition; Joseph Brent Muhlestein: Conceptualization, Investigation, Writing – review & editing, Supervision, Funding acquisition.
