# Supplementary material for: Intermittent fasting and changes in clinical risk scores: Secondary analysis of a randomized controlled trial
Source: Int J Cardiol Cardiovasc Risk Prev. 2023 Sep 11;19:200209. doi: 10.1016/j.ijcrp.2023.200209 (PMC10505676; doi:10.1016/j.ijcrp.2023.200209)
Supplement: Multimedia component 1 [file mmc1.docx]

**Supplemental Materials for the Study:**

**Intermittent Fasting and Changes in Clinical Risk Scores: Secondary Analysis of a Randomized Controlled Trial**

Benjamin D. Horne, PhD, MStat, MPH*†; Jeffrey L. Anderson, MD*‡; Heidi T. May, PhD, MSPH*; Viet T. Le, PA-C, MPAS*§; Tami L. Bair, BS*; Sterling T. Bennett, MD||#; Kirk U. Knowlton, MD*¶; Joseph B. Muhlestein, MD*‡

*Intermountain Medical Center Heart Institute, Salt Lake City, UT; †Division of Cardiovascular Medicine, Department of Medicine, Stanford University, Stanford, CA; ‡Cardiology Division, Department of Internal Medicine, University of Utah, Salt Lake City, UT; §Rocky Mountain University of Health Professions, Provo, UT; ||Intermountain Central Laboratory, Intermountain Medical Center, Salt Lake City, UT; #Department of Pathology, University of Utah, Salt Lake City, UT; ¶Division of Cardiovascular Medicine, Department of Medicine, University of California San Diego, La Jolla, CA.

**Supplemental Table S1**. 26-week changes in the components of the complete blood count and comprehensive metabolic profile. The increase of bilirubin by fasting is well-documented and likely relates to the fasting-induced elevation of fatty acids in the circulation.(S1,S2)

**Intermittent Fasting Ad Libitum Control**

**Parameter 26-week Change 26-week Change p-value**

Red Blood Cell Count (M/μl) 0.070±0.235 0.062±0.208 0.88

Hemoglobin (g/dL) 0.255±0.658 0.115±0.537 0.33

Hematocrit (%) 0.058±5.62 0.546±1.91 0.61

White Blood Cell Count (K/μl) 0.484±1.23 0.036±1.42 0.16

Platelet Count (K/μl) 7.53±24.3 0.76±22.3 0.23

Mean Corpuscular Volume (fL) 0.495±1.53 -0.088±1.95 0.16

MCH (pg) -0.695±4.84 -0.224±0.727 0.19

MCHC (g/dL) -0.058±0.778 -0.200±0.762 0.44

Red Cell Distribution Width* ----- ----- -----

Mean Platelet Volume (fL) -0.013±0.437 -0.012±0.411 0.99

Sodium (mmol/L) -1.21±1.86 -0.58±2.00 0.31

Potassium (mmol/L) -0.208±0.313 -0.118±0.330 0.27

Chloride (mmol/L) -1.63±2.20 -0.97±2.88 0.42

Bicarbonate (mmol/L) -0.290±2.01 0.182±2.63 0.36

Calcium (mg/dL) -0.003±0.34 0.515±2.25 0.84

Glucose† ----- ----- -----

Creatinine (mg/dL) -0.044±0.139 -0.018±0.135 0.42

Blood Urea Nitrogen (mg/dL) -1.32±4.43 -1.03±3.59 0.85

Albumin (g/dL)‡ 0.132±0.201 0.091±0.211 0.29

Bilirubin (mg/dL)‡ 0.218±0.327 0.055±0.246 0.015

Total Protein (g/dL)‡ -1.13±7.90 -0.47±3.47 0.66

ALT (U/L)‡ -0.13±14.2 3.88±10.4 0.18

AST (U/L)‡ 1.71±8.81 2.00±5.61 0.87

ALP (U/L)‡ 4.26±8.49 0.52±9.76 0.27

MCH: mean corpuscular hemoglobin; MCHC: mean corpuscular hemoglobin concentration; ALT: alanine transaminase; AST: aspartate transaminase; ALP: alkaline phosphatase

*As previously published from this trial,(47) 26-week changes in RDW were not different between intermittent fasting and *ad libitum* controls; †As previously published from this trial,(26) glucose was decreased significantly more over the 26 weeks of the trial by intermittent fasting compared to *ad libitum* controls; ‡This parameter was not considered in the derivation of IMRS,(12) but was included in the derivation of ICHRON.(24)

**Supplemental References**

S1. Barrett PVD. Hyperbilirubinemia of fasting. *JAMA* 1971;217(10):1349-1353.

S2. Bloomer JR, Barrett PV, Rodkey FL, Berlin NI. Studies on the mechanism of fasting hyperbilirubinemia. *Gastroenterology* 1971;61(4):479-487.
